# p38*δ* MAPK: Emerging Roles of a Neglected Isoform

**DOI:** 10.1155/2014/272689

**Published:** 2014-09-17

**Authors:** Carol O'Callaghan, Liam J. Fanning, Orla P. Barry

**Affiliations:** ^1^Department of Pharmacology and Therapeutics, Western Gateway Building, Western Road, University College Cork, Cork, Ireland; ^2^Molecular Virology Diagnostic & Research Laboratory, Department of Medicine, Clinical Sciences Building, University College Cork and Cork University Hospital, Cork, Ireland

## Abstract

p38*δ* mitogen activated protein kinase (MAPK) is a unique stress responsive protein kinase. While the p38 MAPK family as a whole has been implicated in a wide variety of biological processes, a specific role for p38*δ* MAPK in cellular signalling and its contribution to both physiological and pathological conditions are presently lacking. Recent emerging evidence, however, provides some insights into specific p38*δ* MAPK signalling. Importantly, these studies have helped to highlight functional similarities as well as differences between p38*δ* MAPK and the other members of the p38 MAPK family of kinases. In this review we discuss the current understanding of the molecular mechanisms underlying p38*δ* MAPK activity. We outline a role for p38*δ* MAPK in important cellular processes such as differentiation and apoptosis as well as pathological conditions such as neurodegenerative disorders, diabetes, and inflammatory disease. Interestingly, disparate roles for p38*δ* MAPK in tumour development have also recently been reported. Thus, we consider evidence which characterises p38*δ* MAPK as both a tumour promoter and a tumour suppressor. In summary, while our knowledge of p38*δ* MAPK has progressed somewhat since its identification in 1997, our understanding of this particular isoform in many cellular processes still strikingly lags behind that of its counterparts.

## 1. p38 Isoform Evolution

The first and now archetypal member of the p38 MAPK family, p38*α* MAPK, was identified by four independent groups in 1994. It was isolated as a 38 kDa protein rapidly tyrosine phosphorylated in response to lipopolysaccharide stimulation [1], as a molecule that binds pyridinyl-imidazole drugs which inhibit the synthesis of proinflammatory cytokines [2] and as an activator of MAPK activated protein kinase 2 (MAPKAP-K2/MK2) and small heat shock proteins in cells stimulated with heat shock or interleukin- (IL-) 1 [3, 4]. This was followed by the subsequent identification of p38*β* MAPK in the same year, p38*γ* MAPK in 1996, and lastly p38*δ* MAPK in 1997 [5–9]. The product of the* S. cerevisiae* HOG1 gene, an important component of osmoregulation and the cell cycle, was found to be a homologue of p38 MAPK [10]. This conservation from yeast to mammals is significant as it indicates that the p38 family is responsible for critical cellular processes. A study of the evolutionary history of MAPKs suggests that each p38 MAPK family member evolved from a single ancestor. In fact it appears that MAPK12 (p38*γ*) arose from a tandem duplication of MAPK11 (p38*β*) on chromosome 22 while MAPK14 (p38*α*) and MAPK13 (p38*δ*) subsequently resulted from a single segmental duplication of the MAPK11-MAPK12 gene unit on chromosome 6 [11]. These gene duplications appear to have occurred before the species separation of nematodes but after the species separation of arthropods. Interestingly, unlike the other p38 isoforms, MAPK13 has not been identified in teleosts [11]. This indicates that a MAPK13 gene deletion event may have occurred subsequent to gene duplication in the evolution of these species. Duplicated genes are generally assumed to be functionally redundant at the time of origin and are eventually silenced. The evolutionary preservation of the four p38 MAPK isoforms therefore suggests functional differentiation of the individual family members. Thus, while the majority of research to date has focused on p38*α* and p38*β* MAPKs, each isoform is an important kinase in its own right with distinct cellular functions. This review aims to highlight components of the previously neglected p38*δ* MAPK signalling pathway and emphasises recent progress in our understanding of p38*δ* MAPK involvement in diverse physiological as well as pathological processes.

The use of pyridinyl-imidazole inhibitors has largely driven the advancement in our understanding of p38*α* and p38*β* MAPK signalling, functions, and substrates. Both p38*α* and p38*β* MAPK are highly sensitive to inhibition by SB203580, SB202190, and newer compounds such as L-167307 [2, 5, 12]. In contrast, the observation that p38*δ* MAPK is insensitive to inhibition by pyridinyl-imidazole compounds has hindered its study in cellular events [7, 9]. The differential sensitivity to these drugs can be attributed to amino acid sequence variability at the ATP binding pocket where these compounds bind competitively, facilitated by interactions with nearby amino acids. Thr106 of p38*α* and p38*β* MAPK has been identified as the major determinant for imidazole inhibitor specificity as it orientates the drug to interact with His107 and Leu108 thereby preventing ATP binding [13]. The equivalent residue in p38*δ* MAPK is a methionine (Met), the large side chain of which prevents binding of these inhibitors. In fact, substitution of Met106 in p38*δ* MAPK with Thr was found to confer some sensitivity to inhibition by SB203580 [14]. Conversely, p38*α* MAPK mutants in which Thr106 is replaced with Met displayed reduced sensitivity to inhibition by SB203580 [15]. It is unfortunate that no potent p38*δ* MAPK specific inhibitor has been identified to date. Although the diaryl urea compound BIRB796 allosterically inhibits p38*δ* MAPK at high concentrations, it is also a powerful inhibitor of p38*α*, -*β*, and -*γ* MAPK [16]. While varying the concentration of BIRB796 and combining it with SB203580 may be of some use in identifying p38*δ* MAPK specific signalling pathways, the possible influence of the other p38 MAPK isoforms, in particular p38*γ* MAPK, must be considered when interpreting any results.

## 2. p38*δ* MAPK Expression and Activation

Unsurprisingly, p38*δ* MAPK shares highly similar protein sequences with the other p38 MAPK isoforms. It displays 61%, 59%, and 65% amino acid identity to p38*α*, -*β*, and -*γ* MAPKs, respectively [7]. Differences in sequence between p38*δ* MAPK and the other p38 MAPK family members can be observed in the ATP binding pocket. This has consequences for inhibitor sensitivity and contributes to substrate specificity. On the other hand, the greatest sequence similarities lie in the highly conserved kinase domains, where the four isoforms share >90% amino acid identity [17]. Within kinase subdomain VIII (of XI), p38*δ* MAPK possesses a TGY dual phosphorylation motif which is the hallmark of p38 MAPKs and is conserved among all known mammalian p38 isoforms [7–9, 18]. p38*δ* MAPK has a distinct distribution profile in human tissue that is relatively limited compared to that of p38*α* and p38*β* MAPK isoforms which are largely ubiquitously expressed [8]. High levels of p38*δ* mRNA have been detected in endocrine tissues such as salivary, pituitary, prostate, and adrenal glands, while more modest levels are expressed in the stomach, colon, trachea, pancreas, skin, kidney, and lung [8]. This differential expression in different cell and tissue types is indicative of a specific biological effect of p38*δ* MAPK activation in these cell types, distinct from that of the other p38 family members.

The murine p38*δ* MAPK amino acid sequence is 92% identical to the human sequence and the adult mouse displays a broadly similar pattern of p38*δ* MAPK expression to that seen in human tissue, that is, lung, testis, kidney, and gut epithelium [18]. Murine p38*δ* MAPK expression varies at different stages in the developing mouse embryo. At 9.5 days it is primarily expressed in the developing gut and septum transversum, while by 15.5 days its expression expands to most developing epithelia [18]. This suggests that p38*δ* MAPK has a role in embryonic development. However, knock-out of p38*δ* MAPK results in mice which are both viable and fertile and exhibit a normal phenotype [19]. Moreover, while p38*β*- and p38*γ*-null mice as well as p38*γ*/p38*δ* double knockout (KO) mice are also phenotypically normal [19, 20], genetic ablation of p38*α* MAPK is embryonic lethal at day 10.5–11.5 [21]. Functional redundancy among the p38 isoforms is a likely explanation with p38*α*, -*β*, or -*γ* compensating for the loss of p38*δ* MAPK activity during development. However, it appears that p38*α* MAPK plays a critical role in early development where its loss cannot be overcome.

**Figure 1:**
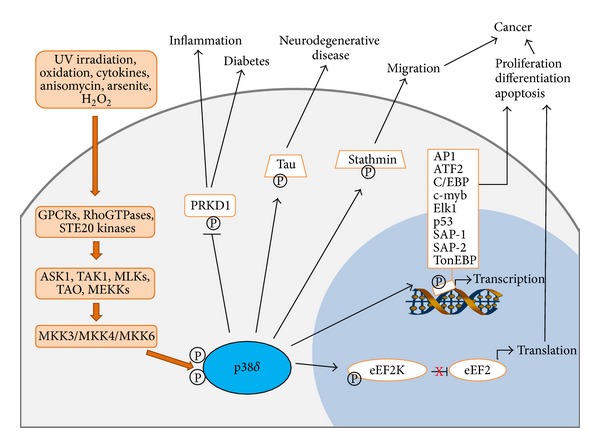
Schematic representation of the current understanding of p38*δ* MAPK signalling and activation. A variety of extracellular stimuli can activate the MAPK signalling pathway resulting in dual phosphorylation of p38*δ* MAPK. Known substrates of active p38*δ* MAPK include transcription factors, structural proteins, kinases, and translation repressors. Phosphorylated substrates affect several cellular processes and contribute to the pathogenesis of diseases such as cancer, diabetes, and neurodegenerative and inflammatory conditions.

p38*α*, -*β*, and -*γ* MAPK isoforms are activated by alterations in the physical and chemical properties of the extracellular environment with diverse triggers including environmental stress signals, inflammatory cytokines, and mitogenic stimuli [1, 5, 6, 22]. Using transiently expressed epitope-tagged p38*δ* MAPK, a similar activation profile has been defined for p38*δ* MAPK [7–9]. It is strongly activated by environmental alterations in osmolarity, ultraviolet (UV) irradiation, and oxidation. It is also moderately activated by chemical stressors and proinflammatory cytokines including arsenite, anisomycin, tumour necrosis factor- (TNF-) *α*, and IL-1. Despite their similar activation profiles, differences in the levels of activation of p38*δ* MAPK and the other p38 MAPK isoforms have been reported. For example, while hyperosmolarity appears to stimulate both p38*α* and p38*δ* to a similar degree, under hypoosmotic conditions, p38*α* MAPK is more strongly activated than p38*δ* MAPK [7]. A range of MAP3Ks have been implicated in the activation of the p38 MAPK pathway, including MLKs (mixed-lineage kinases), TAK1 (transforming growth factor *β* activated kinase 1), ASK1 (apoptosis signal-regulating kinase 1), TAO (thousand-and-one amino acid), DLK1 (dual-leucine-zipper-bearing kinase 1) MEKKs, and ZAK1 (leucine zipper and sterile-*α* motif kinase 1) (Figure 1). To date their individual contribution to p38*δ* MAPK signalling in particular is not yet understood [23–28]. Further upstream of the MAP3Ks is a complex network involving members of the Ras/Rho family of small GTP-binding proteins and heterotrimeric G-protein coupled receptors [29, 30]. This adds to the diversity of signalling from various stimuli contributing to the crosstalk between p38 MAPKs and other signalling pathways.

The upstream direct activators responsible for dual phosphorylation of the p38 MAPK TGY motif are the MAPK kinases (MKKs). p38*δ* MAPK is unique as it can be activated by four separate MKKs: the p38 MAPK specific MKK3 and MKK6 and also the JNK MKKs-4 and -7 [7–9, 18]. However, information regarding the specific contributions of these individual MKKs to p38*δ* MAPK activation in different cell types and under diverse conditions is lacking. Current evidence suggests that activation of p38*δ* MAPK is significantly influenced by both the nature and the strength of the stimulus as well as the cell type involved. This may be the result of varying levels of expression of upstream components of the MAPK signalling cascade in different cell types. For example, MKK3 is the major direct activator of p38*δ* MAPK phosphorylation in response to UV radiation, hyperosmotic shock, and TNF*α* in mouse embryonic fibroblast (MEF) cells [31]. It also appears to be the primary kinase responsible for p38*δ* MAPK activation in response to transforming growth factor-*β*1 (TGF-*β*1) as MKK3 deficiency impairs endogenous p38*δ* activation by TGF-*β*1 in murine glomerular mesangial cells [32]. On the other hand, MKK6 was identified as the major activator of p38*δ* MAPK in KB (HeLa) cells subjected to IL-1, anisomycin, or osmotic stress [9]. Furthermore, MKK7 is reported to be responsible for the activation of p38*δ* MAPK in 293T cells under peroxide stress, mediated by the scaffolding action of islet brain-2 [33]. Further complicating the current understanding of p38*δ* MAPK activation is the likelihood that, in some cases, the cooperation of two MKKs may be necessary. While MKK4 preferentially phosphorylates JNK on Tyr, MKK7 preferentially phosphorylates JNK on Thr [34–36]. Therefore it must be considered possible that the combined activity of these two MKKs may be required to fully phosphorylate p38*δ* MAPK on both the Tyr and the Thr residues. Interestingly, two reports outline a MKK-independent mechanism of activation for p38*α* MAPK via autophosphorylation [37, 38]. While autophosphorylation activity is detected in intrinsically active p38*δ* mutants [39, 40], no such pathway has been observed which activates the endogenous p38*δ* MAPK isoform.

An important factor in determining the biological consequences of p38*δ* MAPK phosphorylation is the strength and duration of the activation signal. p38*δ* MAPK activation is largely transient with activation and downregulation occurring within minutes of stimulation [17]. This is due to the regulatory action of protein phosphatases which again appears to be cell-type specific. While MAPK phosphatase 1 inactivates p38*δ* MAPK in HEK293FT cells, it does not interact with p38*δ* MAPK in the NIH3T3 cell line [41, 42]. The protein serine/threonine phosphatases PP1 and PP2A have also been shown to be involved in p38*δ* MAPK phosphorylation as okadaic acid (OA), a PP1/PP2A inhibitor, causes increased p38*δ* MAPK activity in human epidermal keratinocytes [43].

## 3. Novel p38*δ* MAPK Substrates

While p38 MAPKs are proline-directed kinases, substrate specificity is also determined by docking domains both in the MAPK itself and in the target protein [44]. Therefore, although p38*δ* MAPK substrate specificity overlaps to some extent with that of p38*α*, -*β*, and -*γ* MAPKs, there are a number of notable differences. Common substrates of p38 MAPKs include MBP, PHAS-1, and transcription factors ATF2, SAP1, Elk-1, and p53 (Figure 1). In contrast, however, substrates such as MAPK activated protein kinase 2 (MAPKAP-K2) and MAPKAP-K3 which are the major downstream kinases of p38*α* and p38*β* MAPK are not phosphorylated by p38*δ* MAPK [7–9, 45] (Table 1).

### 3.1. Tau

At the time p38*δ* MAPK was first described the microtubule-associated protein tau was identified as a strong* in vitro* substrate for p38*δ* MAPK [46]. Tau is a component of the cytoskeleton network and under normal conditions it stabilises microtubule assembly by binding to *β*-tubulin. Phosphorylation of tau at T50 by p38*δ* MAPK causes it to be functionally modified and enhances its capacity to promote microtubule assembly. This effect is seen in neuroblastoma in response to osmotic shock where tau T50 phosphorylation occurs soon after p38*δ* MAPK activation, aiding the adaptive response of neurons to changes in osmolarity [46]. It appears, however, that subsequent hyperphosphorylation of tau at additional sites causes it to dissociate from the cytoskeleton, thereby promoting its self-assembly [47]. This aggregation destabilises the microtubule network and contributes to the development of neurofibrillary tangles [48]. Notably, Alzheimer's disease and other neurodegenerative disorders known as tauopathies are characterised by the aggregation in the brain of these neurofilament structures [49]. There is therefore a clearly defined role for p38*δ* MAPK in the pathogenesis of neurodegenerative disease, making it a good potential therapeutic target for these disorders.

### 3.2. Stathmin

There is further evidence of a role for p38*δ* MAPK in cytoskeleton regulation as the microtubule-associated protein stathmin has also been characterised as a good p38*δ* MAPK substrate* in vitro* and in transfected cells exposed to osmotic shock [50]. The normal physiological role of stathmin is to sequester free tubulin and increase depolymerisation of microtubules [51, 52]. It is possible that phosphorylation of stathmin by p38*δ* MAPK blocks its ability to destabilise microtubules and as a result promotes microtubule polymerisation and enhances cell survival under stress conditions.

### 3.3. eEF2K

In response to anisomycin stimulation, p38*δ* MAPK has been shown to be the main p38 MAPK isoform which phosphorylates eukaryotic elongation factor 2 kinase (eEF2K) [53]. Phosphorylation of eEF2K on Ser359 inactivates the kinase and as a result removes its inhibitory phosphorylation of eEF2. eEF2 in turn promotes the movement of the ribosome along mRNA during translation [54]. This suggests that, by inhibiting eEF2K and consequently activating eEF2, p38*δ* MAPK is responsible for driving the translation of proteins associated with stress responses. Consistent with this hypothesis is the observation that the MKK3/6-p38*δ* MAPK-eEF2K pathway in myeloid cells is implicated in the production of the proinflammatory cytokine TNF*α* in bacterial LPS induced acute liver disease [55]. These differences in substrate specificity in combination with its unique tissue distribution profile demonstrate that despite similarities in stimuli the consequences of p38*δ* MAPK can potentially be significantly different to those of the other p38 MAPK isoforms.

## 4. p38*δ* MAPK Function and New Roles in Human Disease

Since the discovery of p38*δ* MAPK in 1997 it has been implicated in a range of diverse physiological events, namely, differentiation, apoptosis, and cytokine production (Figure 1). The greatest understanding of its involvement in these cellular processes has been achieved from work using keratinocytes and the majority of these studies have previously been reviewed [56, 57]. Research is now emerging which establishes p38*δ* MAPK as a regulator of these processes in other cell types. As a result in the past five years p38*δ* MAPK has also been implicated in the pathogenesis of diabetes, inflammatory diseases, and cancer. This progress has been achieved with the development of p38*δ* MAPK KO mouse models which are proving to be a useful tool in elucidating novel roles for p38*δ* MAPK* in vivo*.

### 4.1. Differentiation and Psoriasis

A number of different studies have identified a role for p38*δ* MAPK in keratinocyte differentiation, a process critical for the precise control of normal epidermal homeostasis. p38*δ* MAPK induces keratinocyte differentiation by regulating the expression of involucrin, a marker of keratinocyte terminal differentiation [58–60]. p38*δ* MAPK activation by 12-*O*-tetradecanoylphorbol-13-acetate (TPA), calcium, OA, or green tea polyphenol corresponds with increased involucrin promoter activity, mRNA, and protein expression, as well as increased levels and activity of AP1 and C/EBP transcription factors [58, 59, 61]. Importantly, these responses are observed in the presence of a p38*α*/*β* MAPK inhibitor. In addition p38*γ* MAPK is poorly expressed in keratinocytes [60] confirming a specific role for p38*δ* MAPK. Involucrin expression can also be further upregulated in keratinocytes coexpressing p38*δ* MAPK and PKC*η*, -*δ* or *ε* isoforms [59]. Of note cholesterol-depleting agents and overexpression of MKK6/MKK7 have previously been shown to induce involucrin expression via activation of p38*α* MAPK [60, 62, 63]. This highlights the significance of the stimulus type in determining p38 MAPK isoform activation. A further role for p38*δ* MAPK in keratinocyte differentiation was recently identified. p38*δ* MAPK can regulate expression of ZO-1, an epidermal tight junction membrane protein associated with keratinocyte differentiation [64]. Inhibition of p38*δ* MAPK results in depletion of ZO-1 protein in calcium induced differentiating keratinocytes while other junction proteins remain unaffected [64]. Psoriasis is a benign, chronic inflammatory skin condition that is characterised by hyperproliferation and differentiation of keratinocytes as well as increased expression of inflammatory cytokines. Given the significant role p38*δ* MAPK plays in keratinocyte differentiation, it is no surprise that aberrant p38*δ* MAPK signalling has been implicated in the pathogenesis of psoriasis. Expression of the MAPK13 gene is commonly upregulated in psoriasis [65]. Furthermore, an increase in p38*δ* (as well as -*α* and -*β*) MAPK activity has been detected in psoriatic lesions compared to nonlesional psoriatic skin. After treatment for psoriasis, phosphorylated p38 MAPK levels return to those of uninvolved skin [66].

Further to its role in keratinocyte differentiation p38*δ* MAPK is also implicated in hematopoiesis. In human primary erythroid cells, p38*δ* MAPK mRNA is only expressed in late-stage differentiation where along with p38*α* MAPK it is increasingly activated [67]. This may suggest a functional role for p38*δ* MAPK in erythrocyte membrane remodelling and enucleation. Interestingly, an increase in p38*δ* MAPK mRNA and protein expression is observed as blood monocytes differentiate to macrophages [68]. This suggests a role for p38*δ* MAPK in functions gained by mature macrophages. A possible candidate is phagocytosis given that the microtubule associated protein stathmin is such a strong p38*δ* MAPK substrate.

Most recently, p38*δ* MAPK has been identified as a component of differentiation in bone repair [69]. In bone cell differentiation during wound healing, wild type (WT) monocytes differentiate to calcifying/bone-forming monoosteophils upon treatment with the peptide LL-37. p38*δ* MAPK protein and mRNA is highly expressed in monoosteophils compared to undifferentiated monocytes. Monocytes from p38*δ* MAPK KO mice are incapable of this differentiation, suggesting a critical role for p38*δ* MAPK in this process [69].

### 4.2. Apoptosis and Diabetes

As well as its significant role in keratinocyte differentiation, p38*δ* MAPK has also been identified as a regulator of keratinocyte apoptosis. This dual functional role may be attributed to the overlap of differentiation and apoptosis signalling pathways [70]. As well as inducing involucrin expression [61], OA simultaneously causes disruption of mitochondrial membrane potential and caspase-dependent apoptosis [43]. Overexpression of p38*δ* MAPK enhances this OA driven apoptotic morphology. This response is specific to p38*δ* MAPK activation as it occurred in the presence of the p38*α*/*β* MAPK inhibitor SB203580 [43]. Furthermore, p38*δ* MAPK coexpressed with either MEK6 or PKC*δ*, both upstream p38 MAPK activators, elicited an apoptotic response similar to that induced by OA but in the absence of an external stimulus. This was also independent of SB203580, again ruling out a contribution from other p38 MAPK isoforms [71]. Interestingly, concurrent p38*δ* MAPK activation and inactivation of the proproliferative MAPK ERK1/2 were observed with OA stimulation and PKC*δ*/p38*δ* MAPK coexpression [43, 61, 71]. In fact a reduction in ERK1/2 activation appears to be critical for apoptosis as its constitutive activation inhibited PKC*δ*/p38*δ* MAPK mediated apoptosis [71]. Therefore, it is likely that a specific balance between prosurvival ERK1/2 and proapoptotic p38*δ* MAPK is essential in determining keratinocyte fate. In regulating this balance, p38*δ* MAPK and ERK1/2 form a complex that is translocated to the nucleus upon stimulation by PKC*δ*. This nuclear localisation facilitates ERK1/2 inactivation by nuclear phosphatases, while maintaining p38*δ* MAPK activation [71].

A role for p38*δ* MAPK in apoptosis has recently been demonstrated* in vivo* using p38*δ* MAPK KO mice. Mice deficient in p38*δ* MAPK displayed a fivefold lower rate of pancreatic *β* cell death in response to oxidative stress than WT mice and are afforded protection against insulin resistance induced by a high-fat diet [72]. This would appear to link p38*δ* MAPK to the pathogenesis of diabetes mellitus, a disease characterised by reduced insulin sensitivity and a decrease in insulin-producing pancreatic *β* cells [72]. Increased p38 MAPK pathway activity has indeed been observed in both type 1 and type 2 diabetes and is correlated with late complications of hyperglycemia, including neuropathy and nephropathy [73, 74]. p38*δ* MAPK specifically has also been implicated in the regulation of insulin secretion. Phosphorylation by p38*δ* MAPK negatively regulates the activity of protein kinase D1 (PKD1), a known positive regulator of neuroendocrine cell secretion [72]. Thus, pronounced activation of PKD1 has been observed in pancreatic *β* cells lacking p38*δ* MAPK. As p38*δ* MAPK is normally quite highly expressed in the pancreas this can contribute to heightened insulin secretion and improved glucose tolerance in p38*δ* MAPK-null mice [72]. The pivotal role p38*δ* MAPK plays in integrating insulin secretion and survival of pancreatic *β* cells makes it an attractive potential therapeutic target for the treatment of human diabetes.

### 4.3. Cytokine Production and Inflammatory Diseases

One of the pathways by which p38*α* MAPK was discovered was via its identification as a regulator of proinflammatory cytokine biosynthesis [2]. Thus, its role in cytokine signalling and cytokine-dependent inflammatory diseases is well characterised. Consequently, some recent research using p38*δ* MAPK KO mouse models has focused on identifying specific roles for p38*δ* MAPK in inflammation. A study of p38*δ* MAPK KO mice as well as myeloid-restricted deletion of p38*δ* MAPK in mice has shown that p38*δ* MAPK is required for the recruitment of neutrophils to sites of inflammation [75]. p38*δ* MAPK and its downstream target PKD1 conversely regulate PTEN activity to control neutrophil extravasation and chemotaxis. The accumulation of neutrophils at inflammatory sites is known to trigger inflammation-induced acute lung injury (ALI) which can cause acute respiratory distress syndrome (ARDS), a condition with a high mortality rate [76]. Therefore, abnormal p38*δ*-PKD1 signalling may play an important role in both ALI and ARDS in humans.

Rheumatoid arthritis is a typical example of an inflammatory disease involving chronic synthesis of proinflammatory cytokines which result in synovial hyperplasia and joint destruction [77]. While p38*δ* MAPK (along with -*α*, -*β*, and -*γ*) is expressed in the synovium of rheumatoid arthritis patients its level of activation is lower than that of the four other p38 MAPK isoforms [78]. Despite this low level of activation new research has identified p38*δ* MAPK as an essential component of joint damage in a collagen-induced model of arthritis. p38*γ*/*δ* ^−/−^ mice displayed reduced arthritis severity compared to WT mice [79]. The decrease in joint destruction was associated with lower expression of IL-1*β* and TNF*α* as well as a reduction in T cell proliferation, IFN*γ*, and IL-17 production. Lack of either p38*γ* or p38*δ* MAPK alone yielded intermediate effects, suggesting significant roles for both isoforms in arthritis pathogenesis.

Proinflammatory cytokines also play a significant role in the pathogenesis of inflammatory airway diseases, including asthma, chronic obstructive pulmonary disease (COPD), and cystic fibrosis. While increased mucus production is linked to the morbidity and mortality of such diseases the underlying molecular mechanisms remain somewhat unclear [80]. The critical driver of mucus production is thought to be IL-13 production by immune cells which results in mucin gene expression [81, 82]. In the last few years p38*δ* MAPK has been implicated in the signalling pathway responsible for controlling IL-13 driven excess mucus production. Increased MAPK13 gene expression is evident in the lungs of patients with severe COPD [83]. Novel inhibitors with increased activity against p38*δ* MAPK blocked mucus production by IL-13 in human airway epithelial cells [83]. Thus, in patients with hypersensitivity airway diseases there exists a potential opportunity for therapeutic intervention should specific p38*δ* MAPK inhibitors become clinically available.

## 5. p38*δ* MAPK and Cancer

In recent years, the function of the p38 MAPK signalling pathway in malignant transformation has been intensively studied. As a result, the best characterised isoform, p38*α* MAPK, has been identified as both a tumour promoter [84–86] and a tumour suppressor [87–89]. Recent studies have now also implicated p38*δ* MAPK in cancer development and progression. Like p38*α* MAPK, p38*δ* MAPK would also appear to have both pro- and antioncogenic roles, depending on the cell type studied.

Interest in p38*δ* MAPK as a potential tumour promoter is based on the evidence that p38*δ* MAPK expression and activation are significantly increased in a variety of carcinoma cell lines such as human primary cutaneous squamous carcinoma cells [65], head and neck squamous carcinoma cells and tumours [85], cholangiocarcinoma, and liver cancer cell lines [90]. p38*δ* MAPK was first shown to promote a malignant phenotype (over eight years ago) in head and neck squamous cell carcinoma (HNSCC) [85]. It was shown to regulate HNSCC invasion and proliferation through controlling expression of matrix metalloproteinase-1 and -13 [85, 91]. Moreover the expression of dominant-negative p38*δ* MAPK impaired the ability of cutaneous HNSCC cells to implant in the skin of immunodeficiency mice as well as inhibiting the growth of xenografts [85].

p38*δ* MAPK-null mice have been utilised to demonstrate that p38*δ* MAPK is required for the development of multistage chemical skin carcinogenesis* in vivo*. When compared with WT mice, p38*δ* MAPK-deficient mice displayed reduced susceptibility to 7, 12-dimethylbenz(a)anthracene/12-O-tetradecanoylphorbol-13-acetate induced skin carcinoma with a significant delay in tumour development [92]. Furthermore, both tumour numbers and size were significantly decreased compared with WT mice [92]. This decreased carcinogenesis was associated with reduced levels of proproliferative ERK1/2-AP1 signalling and decreased activation of signal transducer and activator of transcription 3 (Stat3) [92]. The ERK1/2-AP1 pathway is a key cancer promoting cascade previously implicated in skin carcinogenesis [93, 94]. Stat3 meanwhile is an oncogenic transcription factor involved in chemical and UVB-induced transformation [95]. It is also proproliferative and plays a role in angiogenesis and invasion [96, 97]. Therefore p38*δ* MAPK promotion of proliferation via Stat3 may be a significant mechanism in the promotion of carcinogenesis by p38*δ* MAPK. Similarly, p38*δ* MAPK KO mice have reduced susceptibility to development of K-ras driven lung tumorigenesis. Compared with WT mice, p38*δ* ^−/−^/K-RasG12D^+/−^ mice displayed significantly decreased tumour numbers, average tumour volume, and total tumour volume per lung [92]. This is in contrast to p38*α* MAPK-deficient mice which display hyperproliferation of lung epithelium and increased K-Ras-induced lung tumour development [88]. This highlights once again the distinct and often opposing functions of the individual p38 MAPK isoforms.

Further evidence for a specific role of p38*δ* MAPK in promoting cancer progression has most recently been demonstrated in cholangiocarcinoma (CC) [90]. p38*δ* MAPK expression is upregulated in CC when compared with normal biliary tract tissue. Knockdown, however, of p38*δ* MAPK expression by siRNA transfection significantly inhibited motility and invasiveness of CC cells. In contrast, overexpression of p38*δ* MAPK in these cells results in enhanced invasive behaviour. Significantly, p38*δ* MAPK may prove to be a useful marker for the differential diagnosis of CC over hepatocellular carcinoma where it lacks expression [90].

In contrast to the relatively well characterised role of p38*δ* MAPK as a tumour promoter an increasing number of reports since 2011 outline its activity as a tumour suppressor. The first indication of a tumour suppressive role for p38*δ* MAPK was observed in mouse embryonic fibroblasts (MEF). p38*δ* ^−/−^ (and p38*γ* ^−/−^) MEFs displayed increased cell motility compared to WT cells [98]. Furthermore, while WT fibroblasts ceased to proliferate after reaching 100% confluency, p38*δ* ^−/−^ MEFs continued to grow, forming foci rather than a monolayer [98]. This deregulation of contact inhibition is significant as it is a hallmark of malignant transformation [99]. Our own recent studies have also identified a role for p38*δ* MAPK in the control of oesophageal squamous cell carcinoma (OESCC) migration, invasion and contact inhibition, processes which are crucial for the progression of primary tumours to distant metastases [100]. Reintroduction of p38*δ* MAPK into OESCC cells which lack endogenous expression significantly impaired cell proliferation, migration, and invasion as well as significantly reducing the number of colonies formed on soft agar compared to WT. These effects were further enhanced in cells transfected with a constitutively active form of p38*δ* MAPK [100]. Furthermore, p38*δ* MAPK expression appears to influence the chemosensitivity of OESCC to apoptosis. Our recent study indicates that OESCC cells expressing p38*δ* MAPK are significantly more sensitive to cisplatin and 5-fluorouracil combination therapy than p38*δ* MAPK-deficient cells [101]. These findings are significant as they suggest that p38*δ* MAPK may be a useful predictor of response to chemotherapy in OESCC patients. Further supporting the hypothesis that loss of p38*δ* MAPK confers a survival advantage, p38*δ* MAPK expression was found to be downregulated in brain metastases of triple-negative breast cancer (TNBC). Abolition of p38*δ* MAPK expression in TNBC induced cell growth, while overexpression of p38*δ* MAPK in brain metastases reduced growth rates [102].

Cancer genomes are increasingly associated with epigenetic alterations whereby tumour suppressor genes exhibit promoter hypermethylation. Interestingly, hypermethylation of the MAPK13 gene promoter region has recently been characterised in both malignant pleural mesothelioma [103] and primary cutaneous melanoma [104]. This methylation is associated with downregulation of p38*δ* MAPK mRNA and protein expression. Melanoma cell lines displaying MAPK13 gene promoter methylation do not express significant levels of p38*δ* MAPK when compared to fibroblasts, melanocytes, and melanoma cell lines with unmethylated MAPK13 promoters. Furthermore, treatment of melanoma cells with the demethylating agent 5-aza-2′-deoxycytidine significantly increases the expression of the MAPK13 gene [104, 105]. Importantly, reestablishment of p38*δ* MAPK expression in melanoma cells with MAPK13 hypermethylation suppresses cell proliferation. The effect was further enhanced upon expression of a constitutively active form of p38*δ* MAPK. Interestingly, however, overexpression of p38*δ* MAPK or its constitutively active form in cells in which MAPK13 was not epigenetically silenced only marginally affected proliferation [104].

## 6. Conclusions and Future Directions for p38*δ* MAPK Research

p38*δ* MAPK is a unique stress-responsive protein kinase. It is mainly activated by environmental stresses, including UV radiation, osmotic shock, and oxidative stress, to illicit an adaptive response within the cell. This is mediated through phosphorylation of substrates involved in cytoskeleton organisation such as tau and stathmin, as well as transcription factors responsible for the expression of stress-responsive genes [106, 107]. Since the discovery of the p38 MAPK family in the mid-nineties they have increasingly been associated with cellular processes such as proliferation, differentiation, development, apoptosis, and migration [108]. Research to date has generally focused on the first two isoforms to be discovered. It is now becoming increasingly clear however that conclusions drawn from p38*α* MAPK (and to an extent p38*β* MAPK) studies cannot be automatically applied to the p38*γ* and p38*δ* MAPK isoforms due to their different expression patterns, substrate specificities, and sensitivity to chemical inhibitors. Studies carried out in the last few years have led to some small advances in our knowledge of the regulation of p38*δ* MAPK and its physiological roles. In particular, the development of p38*δ* MAPK KO mouse models has yielded a greater understanding of the consequences of p38*δ* MAPK signalling* in vivo*. Roles for p38*δ* MAPK in important cellular processes such as differentiation and apoptosis have been identified [69, 72]. As a result, p38*δ* MAPK is now implicated in a variety of pathological conditions including inflammatory diseases, diabetes, and cancer [72, 75, 92, 98]. Most importantly, p38*δ* MAPK may now be considered as a potential therapeutic target for the treatment of these disorders.

The implication of p38*δ* MAPK in a wide range of human diseases should strengthen future research interest in this isoform. The main limiting factors to the further study of p38*δ* MAPK functions, however, are the lack of specific inhibitors and activators. Fuelled by the prospect of therapeutic benefit for patients with diabetes or inflammatory disease, for example, the search for more potent and specific inhibitors of p38*δ* MAPK is ongoing [83]. These may not only provide potential treatments for the conditions outlined here but could also afford us the opportunity to delineate specific p38*δ* MAPK functions in the absence of involvement from other p38 MAPK isoforms. This in turn may identify other diseases where p38*δ* MAPK could be a potential therapeutic target. In this review we also present important and interesting observations which suggest that focusing on identification of specific p38*δ* MAPK activators is also warranted. This may in the future translate to the development of novel therapeutic strategies for patients with OESCC or melanoma. Whether considering the possible therapeutic benefits of p38*δ* MAPK inhibitors or activators it is important to heed the diversity and important role(s) of p38*δ* MAPK signalling in normal physiological processes. In conclusion, uncovering some of the physiological as well as pathological roles of p38*δ* MAPK since its discovery almost twenty years ago has been somewhat successful. However, based on our current knowledge continued focused research on this particular isoform is necessary if p38*δ* is to translate into a novel therapeutic target for a range of diverse human diseases.

## Figures and Tables

**Table 1:** Known p38*δ* MAPK substrates and their biochemical functions.

Substrate	Function	Consequences of phosphorylation
AP1	Transcription factor	Activation of transcription, involucrin expression, keratinocyte differentiation [58]
ATF2	Transcription factor	Activation of transcription [7, 9]
C/EBP	Transcription factor	Keratinocyte differentiation [59]
c-myb	Transcription factor	c-myb degradation [109]
eEF2K	Inhibitory kinase	eEF2 activation, protein synthesis [53]
Elk1	Transcription factor	Activation of transcription [7, 9]
p53	Transcription factor	p21 expression, G_1_ phase arrest [9, 110]
PHAS-1	Translation repressor	Dissociation from eIF4E, activation of translation [7]
PRKD1	Serine-threonine kinase	Inhibition of PRKD1 activity [72]
SAP-1	Transcription factor	Activation of transcription [9]
SAP-2	Transcription factor	Activation of transcription [9]
Stathmin	Microtubule protein	Cytoskeleton reorganisation [50]
Tau	Microtubule protein	Microtubule assembly, tau self-aggregation [46]
TonEBP/OREBP	Transcription factor	Impaired TonEBP/OREBP transcriptional activity [41]

AP1: activator protein 1; ATF2: activating transcription factor 2; C/EBP: CCAAT (cytosine-cytosine-adenosine-adenosine-thymidine)-enhancer-binding protein; myb: myeloblastosis; eEF2K: eukaryotic elongation factor 2 kinase; eEF2: eukaryotic elongation factor 2; PHAS-1: phosphorylated heat- and acid-stable protein 1; PRKD1: protein kinase D 1; SAP: serum response factor accessory protein; eIF4E: eukaryotic translation initiation factor 4E.

## Abbreviations

UV: Ultraviolet
GPCR: G-protein coupled receptor
GTP: Guanine tyrosine phosphatase
STE20: Sterile 20
ASK1: Apoptosis signal-regulating kinase 1
TAK1: Transforming growth factor *β* activated kinase 1
MLK: Mixed-lineage kinase
TAO: Thousand-and-one amino acid
MEKK: Mitogen activated protein kinase kinase kinase
MKK: Mitogen activated protein kinase kinase
PRKD1: Protein kinase D 1
AP1: Activator protein 1
ATF2: Activating transcription factor 2
C/EBP: CCAAT (cytosine-cytosine-adenosine-adenosine-thymidine)-enhancer-binding protein
myb: Myeloblastosis
SAP: Serum response factor accessory protein
eEF2K: Eukaryotic elongation factor 2 kinase
eEF2: Eukaryotic elongation factor 2
PHAS-1: Phosphorylated heat- and acid-stable protein 1
eIF4E: Eukaryotic translation initiation factor 4E.
